# The effect of a pharmacist consultation on pregnant women’s quality of life with a special focus on nausea and vomiting: an intervention study

**DOI:** 10.1186/s12884-020-03472-z

**Published:** 2020-12-09

**Authors:** Maria Bich-Thuy Truong, Elin Ngo, Hilde Ariansen, Ross T. Tsuyuki, Hedvig Nordeng

**Affiliations:** 1 Pharmacoepidemiology and Drug Safety Research Group, Department of Pharmacy, University of Oslo, Oslo, Norway; 2 The Norwegian Pharmacy Association, Oslo, Norway; 3 Department of Pharmacology, Faculty of Medicine and Dentistry, University of Alberta, Edmonton, Canada; 4 Department of Child Health and Development, Norwegian Institute of Public Health, Oslo, Norway

**Keywords:** Pharmacist intervention, Pregnancy, Quality of life, Medications, Nausea and vomiting in pregnancy, Community pharmacy

## Abstract

**Background:**

Maternal wellbeing and quality of life (QOL) are increasingly being recognized as important for healthy pregnancies. The aim of this study was to investigate the impact of a pharmacist consultation on pregnant women’s QOL focusing on nausea and vomiting in pregnancy (NVP), and patient satisfaction.

**Methods:**

For this intervention study in 14 community pharmacies, women in early pregnancy were recruited and assigned to a pharmacist consultation (intervention) or standard care (control). The consultation aimed to address each woman’s concerns regarding medications and pregnancy-related ailments. Data were collected through online questionnaires at baseline (Q1) and during the second trimester (Q2). The intervention group completed an additional satisfaction questionnaire after the consultation was completed. The primary outcome was the impact of the intervention on the Quality of Life Scale (QOLS) scores between the first and second trimesters. The impact of the intervention was assessed by linear regression, and secondary analyses were performed to assess effect modification by NVP.

**Results:**

Of the 340 women enrolled in the study, we analyzed data for 245. Half (170/340) of the original participants were allocated to the intervention group, of whom 131 received the pharmacist consultation. Most women (75%, 78/96) reported that the consultation was useful to a large/very large extent. The consultation had no overall impact on QOLS scores between the first and the second trimesters compared with standard care (adjusted β: 0.7, 95% CI: -2.1, 3.4). The impact of the intervention on QOLS was greater amongst women with moderate/severe NVP (adjusted β: 3.6, 95% CI: -0.6, 7.7) compared to those with no/mild NVP (adjusted β: -1.4, 95% CI: -5.1, 2.2) (interaction term study group*NVP severity, *p* = 0.048).

**Conclusions:**

The pregnant women highly appreciated the pharmacist consultation, but the intervention did not affect their QOL scores compared with standard care. Future studies should further explore the effect of a pharmacist consultation specifically for NVP and on other outcomes such as use of health care services and medication use in pregnancy.

**Trial registration:**

Retrospectively registered in ClinicalTrials.gov (identifier: NCT04182750, registration date: December 2, 2019).

**Supplementary Information:**

The online version contains supplementary material available at 10.1186/s12884-020-03472-z.

## Background

The aim of antenatal care is to ensure maternal and fetal wellbeing throughout the pregnancy. This includes providing information about health and lifestyle and detecting and resolving potential complications. Maternal wellbeing, mental health, and quality of life are increasingly being recognized as important for healthy pregnancies. Risk factors for poor quality of life during the gestational period include a range of common pregnancy-related ailments, such as heartburn, fatigue, sleeping problems, and nausea and vomiting in pregnancy (NVP) [1]. Healthcare providers in antenatal care should educate pregnant women about how to self-manage pregnancy-related ailments to improve their quality of life. A particular focus should be to target NVP because even mild symptoms have been shown to significantly reduce pregnant women’s quality of life [2–4]. More specifically, studies have shown that NVP can have a considerable impact on daily functioning, desire to become pregnant again, and the ability to care for other children [2].

NVP is one of the most common pregnancy-related ailments and affects up to 90% of all pregnancies [5], with symptoms ranging from mild to severe. Because NVP often occurs in early pregnancy, before the first antenatal care check-up, management of NVP relies on self-care and potentially on advice from primary healthcare providers such as community pharmacists. There is a general perception that NVP is a natural state of pregnancy, and the condition has consequently often been mismanaged or left untreated [3]. The term “morning sickness” may also be misleading because the symptoms often are present throughout the day. Although the symptoms usually resolve spontaneously by gestational week 16, from 5 to 10% of women experience prolonged symptoms, sometimes throughout the entire pregnancy [6]. Early recognition of NVP and initiation of optimal management are essential and may prevent further complications, such as dehydration, ketonuria, excessive weight loss, malnutrition, and in worst cases, the diagnosis of hyperemesis gravidarum [7]. Considering the high prevalence of NVP and the major discomfort it can entail, optimally managed NVP may be of great importance in improving women’s health during pregnancy.

Pregnant women have a profound need for health information to make the safest treatment decisions for themselves and the fetus [8–10]. Prior studies have shown that between 46 and 77% of pregnant women need medication information [9] and that they trust healthcare providers, including pharmacists, to provide them with reliable information about medications in pregnancy [9, 11, 12]. A recent feasibility study showed that women report the need for general information about medications during pregnancy and treatment of pregnancy-related ailments [13]. Although healthcare providers are considered trusted information sources, up to 96% of pregnant women seek health information on the internet [14, 15]. Pregnant women tend to engage in online discussion forums while waiting for their clinical appointment [16], or when their healthcare providers fail to provide sufficient information to meet their needs [17]. Earlier studies have also demonstrated that pharmacists have an important role in promoting peri-conceptional use of folic acid supplements [18], improving asthma control and inhaler technique among pregnant women [19], and identifying and solving medication-related problems for obstetric patients in secondary care [20, 21]. Community pharmacists have the skills and knowledge and are optimally placed in the community to provide medication information and empower pregnant women to make safe treatment decisions. This is especially important during the first trimester when antenatal care usually is not yet established.

Although pharmacist interventions have shown promising outcomes for certain obstetric patients and several other non-pregnant patient populations [22], no studies have investigated the effect of a community pharmacist intervention in early pregnancy on women’s quality of life [23, 24].

## Methods

### Aim

The primary aim of this study was to investigate the impact of a pharmacist consultation in early pregnancy on women’s quality of life between the first (baseline) and second trimesters. The secondary aim was to assess whether NVP could modify the effect of the intervention on changes in quality of life during this period. We also assessed patient satisfaction with the pharmacist consultation.

### Study design and sample size

This is a project within the SafeStart study, an assigned 1:1 intervention study conducted by 15 pharmacists in 14 community pharmacies in Norway. Community pharmacists in Norway have either a Bachelor of Pharmacy (3-year education) or a Master of Pharmacy (5-year education). Holders of both degrees are licensed as pharmacists, and both can perform the same tasks in the community pharmacy (e.g., dispensing medications and advising patients on how to use their medication safely and efficiently). However, only a Master of Pharmacy can be a pharmacy manager. All community pharmacists have dedicated working hours to maintain and develop their professional skills.

The recruitment of study pharmacists to the SafeStart study was led and coordinated by the Norwegian Pharmacy Association and based on voluntary participation. All four main pharmacy chains (Apotek 1, Boots apotek, Vitusapotek, and Sykehusapotekene) in Norway participated. The study pharmacies had a wide geographical distribution and were mainly located in urban and suburban areas (Additional File 1).

The aim of the SafeStart study was to investigate whether a pharmacist consultation provided in early pregnancy can affect pregnant women’s quality of life, need for sick leave, self-management of pregnancy-related ailments, and their use of healthcare services, and whether the pharmacist consultation can be cost-effective and implemented in primary care. We estimated that a total sample size of 385 pregnant women would be needed to detect a clinically significant difference of 5 points in the change of the Quality of Life Scale (QOLS) score from the first (baseline) to the second trimester between the two study groups [2, 25]. The sample size estimation was based on a two-sided α of 0.05, 80% power, and a dropout rate of 30%. The SafeStart study was conducted according to the CONSORT guidelines [26].

### Study population, setting, and assignment to study groups

Participants were recruited through social media and posters in the local areas of the 14 study pharmacies from February 2018 to February 2019. All pregnant women (≥ 18 years) in their first trimester, independent of comorbidities, were eligible for inclusion. All recruiting methods referred the potential participants to the study website where they could enroll by signing the online consent form with an electronic ID. The electronic ID is unique for every inhabitant in Norway, and it is routinely used to access own personal information, e.g., health and financial information. Women had to confirm that they met the inclusion criteria in the online consent form before they were enrolled in the study. A data preprocessing system (ADPS) automatically handled the online enrollment of participants, distribution of online questionnaires, and assignment of participants to one of the study groups. Every other participant enrolled in the study was allocated to the intervention group. The ADPS has been described previously [13].

### Intervention

#### Training of study pharmacists

All study pharmacists completed a training program, including three e-courses about pharmacotherapy in pregnancy; self-study of a compendium covering common pregnancy-related ailments, including NVP; a study manual covering relevant aspects of a clinical consultation; and a full-day workshop focusing on clinical and risk communication. The workshop also specifically focused on the standardization of the NVP consultation, i.e., how to give consultation about both pharmacologic and non-pharmacologic treatment based on the women’s NVP severity (described in more detail below).

#### Consultation definition and logistics

The intervention was defined as *“A planned, structured, and individualized consultation with the purpose of relieving pregnant women of any concern and answering questions they may have regarding ailments and medication use during pregnancy.”* The consultations had a timeframe of approximately 15 min and were conducted in the pharmacy’s private information room or over the telephone. Participants allocated to the intervention group specified the date, time, and their pharmacy preference for the consultation and whether they wanted to have it at the pharmacy or over the telephone. The participants received an encouragement to book the consultation as soon as possible after enrollment and preferably during the first trimester. After receiving a booking, the study pharmacists contacted the participant to confirm the booking or suggest a new time.

#### Performing the consultation

The consultations were performed in a patient-centered manner, focusing on each woman’s needs independent of occurring diseases. The consultation was divided into an introduction, middle, and closing. The study pharmacists received a study manual describing each part, with examples of relevant wording and questions. A consultation guide has previously been published [13]. In short, the introduction focused on getting to know the participant and setting the parameters for the consultation (content and time). The study pharmacists had access to each participant’s baseline characteristics, but every consultation began with a question for the participant about her concerns and needs. The middle part of the consultation focused on exploring and addressing the participant’s concerns and needs. The closing part was specifically used to make sure that the women could ask any last questions and for the pharmacists to ensure that the women had captured the key points from the consultation. The pharmacists documented each consultation on a structured form.

#### The NVP consultation

Information about NVP was based on each woman’s needs and symptom severity according to national recommendations (Additional File 2). NVP severity was categorized as mild, moderate, or severe according to the Pregnancy-Unique Quantification of Emesis 24 (PUQE) score (described in more detail below). In brief, women with mild NVP were advised to try non-pharmacologic measures, including dietary and lifestyle changes, ginger, and acupressure. Women with moderate/severe NVP were informed about safe antiemetic treatment. All women with NVP, regardless of severity, received information about the importance of adequate hydration. All women were also informed that there are safe antiemetic treatment options available and encouraged to reach out again if NVP symptoms worsened.

### Control group

Women allocated to the control group received a general email about the study after the completion of the baseline questionnaire and followed standard antenatal care.

### Data collection

The data in the SafeStart study are collected through four online questionnaires, from early pregnancy to 3 months post-partum. For this study, we used data from the two first questionnaires (Q1 and Q2), for which data collection was completed. Participants completed Q1 immediately after study enrollment in the first trimester and completed Q2 during the second trimester (13 weeks after enrollment). The timing of Q2 was based on prior studies on quality of life in pregnancy [27–29]. Women in the intervention group completed an additional satisfaction questionnaire after the consultation. All questionnaires were automatically distributed by the ADPS to the participants via email.

### Outcome measures

#### Change in quality of life

The primary outcome of this study was the difference in change in quality of life from baseline (Q1) to the second trimester (Q2) between groups, as measured by the QOLS [30]. This quality of life instrument was chosen because it has previously been used to measure quality of life among pregnant women with NVP in Norway [2]. The QOLS was originally developed in English and consists of 16 questions addressing a respondent’s current level of satisfaction regarding material and physical wellbeing, relationship with other people, sociability, community and civic activities, personal development and fulfillment, and recreation [30]. The questions are answered on a seven-point Likert scale, for 1–7 points possible for each question. The total score range is 16 to 112, and a higher score indicates better quality of life. The QOLS scores for the general female population and females experiencing diseases or health problems in Norway are 85 ± 12.3 and 81 ± 12.8, respectively [25]. A previous Norwegian study found average QOLS scores among women with NVP as follows: mild NVP = 73, moderate NVP = 67, severe NVP = 63 [2]. The validated Norwegian version of the QOLS used in this study has been found to have a satisfactory test-retest reliability (0.83) and internal consistency reliability (Cronbach’s alpha: 0.86 at time 1 and 0.89 at time 2) [31].

#### Women’s satisfaction with the consultation

Women who received the consultation were provided a link to the online satisfaction questionnaire immediately afterward. The questionnaire contained seven questions about how satisfied the participant felt and the extent to which she found the consultation useful (very little/little/neutral/large/very large extent). The questionnaire also included the questions, “Would you like the pharmacist consultation again in a future pregnancy? (yes/no/I do not know”, “What did you like the most about the pharmacist consultation?” and “State three things you would like to improve with the pharmacist consultation.” The two latter questions were optional and answered as free text entries.

### Other measures

#### NVP

The severity of NVP was determined by using the PUQE score [32]. The PUQE was administered to all participants at baseline (Q1, first trimester) and to women with NVP during the pharmacist consultations. The PUQE score was originally developed in English to assist clinicians and pregnant women in assessing the severity of NVP and to determine the appropriate treatment. It consists of three questions about nausea, vomiting, and retching symptoms during the past 24 h. Each item gives a score of 1–5, adding up to a total score ranging from 3 to 15. The total score categorizes NVP severity over the previous 24 h as mild (≤ 6), moderate (7–12), or severe (≥ 13) [32–34]. The mild NVP group also included women who did not have NVP because the PUQE does not distinguish between no NVP (PUQE = 3) and mild NVP (PUQE ≤ 6). Here, we designate this group as “No/mild NVP”. In their validation study, Koren et al. found that an increased PUQE score was significantly associated with discontinuation of prenatal vitamins, increased hospitalization and healthcare cost, and reduced maternal wellbeing [32]. The translated Norwegian version used in this study has been validated as a robust indicator of severe hyperemesis gravidarum and insufficient nutritional intake in pregnancy [33].

#### Maternal background characteristics

At baseline, participants completed the Q1 and reported maternal background characteristics, including age, area of residence, highest level of education, marital status, and occupation. They were also asked to report lifestyle characteristics (smoking status before and/or during pregnancy and alcohol consumption after awareness of pregnancy), and pregnancy-related background characteristics (gestational age, parity, infertility treatment, and use of folic acid before and during pregnancy). Maternal characteristics were also obtained from other birth cohorts in Norway to assess the representativeness of our study population.

#### Health-related characteristics and medication use

Participants were presented with a list of eight common pregnancy-related ailments: NVP, nasal congestion/common cold, heartburn and reflux problems, congestion, urinary tract infection, sleeping problems, headache, and pain in the neck, back, or pelvic girdle. The participants who selected “Yes” for having experienced any of these ailments during pregnancy were asked to report any related medication used for each. Participants were also presented with a list of nine common chronic conditions (asthma, allergy, hypothyroidism, depression/anxiety, coronary diseases, rheumatic conditions, epilepsy, and diabetes) and asked to report any medication use for the chronic conditions. The participants could also report as free text on other pregnancy-related ailments and/or chronic conditions that were not listed.

### Statistical analyses

Categorical baseline characteristics (parity, education, occupation, marital status, chronic condition, NVP severity, infertility treatment, use of folic acid, smoking status, and alcohol in pregnancy) are summarized as the proportion of participants in each group. Continuous variables are summarized as the mean with standard deviation (SD) and range (gestational weeks, maternal age, PUQE, and QOLS). Differences in baseline characteristics between the two study groups were assessed with the Chi-square test for categorical variables and with the independent Student’s t-test for continuous variables. All results with *p* < 0.05 were considered statistically significant.

The primary analyses assessing the impact of the pharmacist consultation on women’s change in QOLS scores between Q1 and Q2 were performed using univariate and multiple linear regressions. The assumptions for data normality were tested and found to meet the criteria. The adjusted analyses included the QOLS score at baseline only because none of the other baseline characteristics significantly differed between the two study groups (i.e., all *p* ≥ 0.05). The results are presented as the crude and adjusted changes in QOLS (β coefficients) between the intervention and control groups with the 95% confidence interval (95% CI). Because NVP can affect pregnant women’s quality of life, secondary stratified analyses were performed to assess the effect modification by NVP (classified as no/mild or moderate/severe as defined by the PUQE score). To formally test the difference in the effect estimates (β coefficients) in the stratified analyses, a linear regression model was fitted by including the interaction term between study group (intervention or control) and NVP classification (no/mild or moderate/severe). The predicted mean changes in QOLS by this model are presented as an interaction plot. The primary and secondary stratified analyses were performed according to the intention-to-treat principle (*n* = 245), i.e., including all women who responded to both Q1 and Q2, and allocated to one of the study groups (all women allocated to the intervention group independent if they completed the pharmacist consultation or not).

Sensitivity analyses were performed according to the per-protocol principle (*n* = 229), i.e., restricted to women who responded to both Q1 and Q2, and adhered to their allocation group (i.e., only women who completed the pharmacist consultation if allocated to the intervention group). Non-responders to either questionnaire (Q1 or Q2) were excluded from all analyses. All analyses were done using Stata/MP v.15.1.

## Results

### Study population

**Fig. 1:**
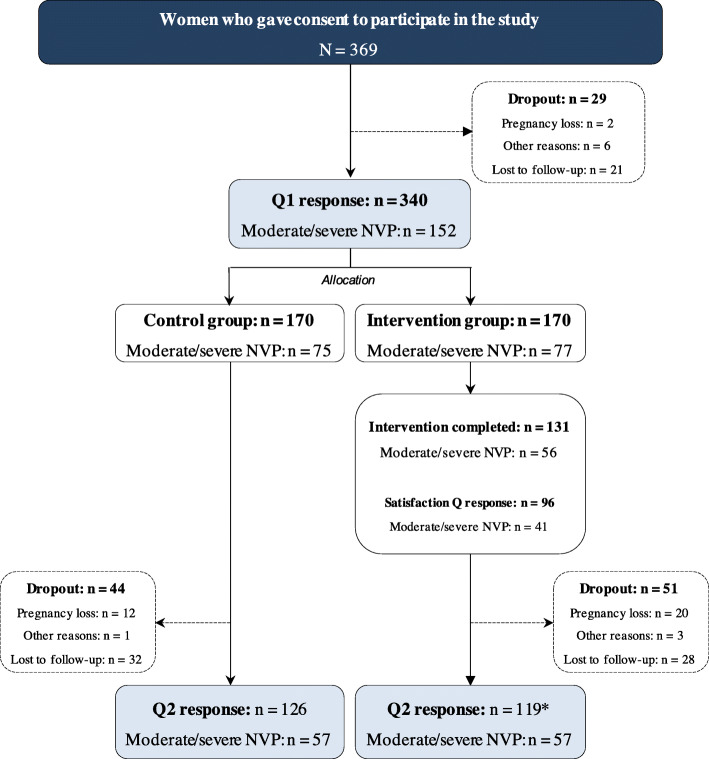
Flowchart of the total study sample, including dropouts. *16 participants responding to the second questionnaire (Q2) in the intervention group did not complete the intervention. The total number of participants in the intervention group included in the per-protocol analyses was 103. Abbreviations: *NVP* nausea and vomiting in pregnancy; *Q1* baseline questionnaire in the first trimester

A total of 369 women consented to participate in the study (Fig. 1). Among these, 340 responded to the baseline questionnaire and were allocated to either the intervention group (*n* = 170) or the control group (*n* = 170). Comparison of baseline characteristics of the analyzed sample according to the intention-to-treat principle (*n* = 245) showed that the characteristics did not statistically differ between the groups (Table 1). The analyses according to the per-protocol principle included 229 participants. The Q1 and Q2 were filled out on average during gestation week 7.4 ± 2.2 and 21 ± 2.6, respectively, whereas the average consultation was performed during gestation week 8.9 ± 2.7.

**Table 1:** Comparison of baseline characteristics

Baseline characteristics	Intervention group^*^ n = 119	Control group^*^ n = 126	General birthing population in Norway/other studies
Gestation week, mean (SD, range)^a^	7.6 (2.1, 3–12)	7.5 (2.3, 3–12)	–
Maternal age, mean (SD, range)	31.3 (4.0, 21–41)	31.1 (4.6, 18–44)	31.0 (4.9)^c^
Primiparous, %	58.8	48.4	42.4^c^
University/college degree, %	82.4	83.3	51.5^d^
Employed, %	89.9	81.0	86.4^e^
Married/cohabitating, %	97.5	96.0	93.6^c^
≥ 1 chronic condition, %	42.9	46.0	–
PUQE score^b^, mean (SD, range)	6.4 (2.8, 3–14)	6.1 (2.5, 3–15)	–
Moderate or severe NVP^b^, %	47.9	45.2	91.2^f^
Infertility treatment in current pregnancy, %	10.9	16.7	3−4^g^
Folic acid supplement before and during pregnancy, %	97.5	98.4	33.8/79.1^c,h^
Smoking in pregnancy, %	1.7	0.8	3.4^c^
Alcohol in pregnancy, %	5.0	3.2	4.1^i^
QOLS, mean (SD, range)	89.0 (12.5, 42–112)	91.1 (10.2, 62–112)	81 (12.8)^f^

Comparison of baseline characteristics of the study participants according to the two study groups. Characteristics of the general birthing population of Norway from other sources are also presented

Abbreviations: *SD* standard deviation; *NVP* nausea and vomiting in pregnancy; *QOLS* Quality of Life Scale

^*^The Chi-Squared test was used to compare categorical variables between the two study groups. The two-sided Students t-test were used to compare the continuous variables (gestation week, maternal age, PUQE, and QOLS). No results indicated statistical significance (all *p* ≥ 0.05)

^a^Total number intervention group, *n* = 118; total number control group, *n* = 124. Three women with unknown gestation week at baseline

^b^NVP severity based on the Pregnancy-Unique Quantification of Emesis (PUQE) score: mild ≤ 6; moderate 7–12; severe ≥ 13

^c^Data from the Norwegian Medical Birth Registry for 2018

^d^Data from Statistics Norway, women aged 20–39 in 2018

^e^Data from Statistics Norway, women aged 25–39 in 2018

^f^Heitmann et al. [2]

^g^Data from the Norwegian Biotechnology Advisory Board

^h^Folic acid supplement before/during pregnancy

^i^Mårdby et al. [35]

A total of 188 women (188/340, 55.3%) had no or mild NVP at baseline (PUQE ≤ 6), whereas 152 women (152/340, 44.7%) had moderate or severe NVP (PUQE = 7–15). A flowchart including the numbers of women with NVP is depicted in Additional File 3. A total of 124 participants (124/369, 33.6%) dropped out of the study. Of these, 27.4% (34/124) dropped out because of pregnancy loss (Fig. 1). The baseline characteristics of the analyzed sample and those who dropped out did not differ materially (Additional File 4).

### Intervention

Of 170 participants allocated to the intervention group, 131 completed the pharmacist consultation. Just over a third (46/131, 35.1%) were performed at the pharmacy, and the remaining consultations (85/131, 64.9%) were performed over the telephone. The average consultation was performed in gestation week 8.9 ± 2.7 (range: 4–22), and the median duration was 17 min (SD: 6; range: 4–40 min). Most of the women (107/131, 81.2%) wanted information about NVP and NVP management. Most participants who responded to the satisfaction questionnaire (96/131, 73.3%) found the consultation useful to a large/very large extent (Table 2). More than half of the respondents stated that they would want the consultation again in a future pregnancy (61/96, 63.5%). The free text entries revealed that the women especially found it useful to receive information tailored to their needs and to get information and reassurance about safe medication use in pregnancy. These responses also indicated that the women wanted more time during the consultation and more supplemental materials with general information about the pregnancy. Satisfaction scores (Table 2) did not differ (all *p* > 0.05) between women with no/mild and women with moderate/severe NVP (Additional File 5).

**Table 2:** Participant satisfaction with the pharmacist consultation

	Very little	Little	Neutral	Large	Very large
	n (%)	n (%)	n (%)	n (%)	n (%)
In total, to what extent are you satisfied with the consultation you were provided?	0 (0.0)	1 (1.0)	6 (6.3)	42(43.8)	47 (49.0)
To what extent did you find the consultation useful?	1 (1.0)	3 (3.1)	14 (14.6)	50 (52.1)	28 (29.2)
To what extent was the consultation worth the time spent?	1 (1.0)	3 (3.1)	12 (12.5)	47 (49.0)	33 (34.4)
To what extent did you find a solution to your problems/concerns?	14 (14.6)	2 (2.1)	15 (15.6)	43 (44.8)	22 (22.9)
To what extent was the pharmacist concerned about you and your pregnancy?	0 (0.0)	1 (1.0)	12 (12.5)	31 (32.3)	52 (54.2)
To what extent has the consultation given you better insight into how to use medications during pregnancy?	2 (2.1)	3 (3.1)	20 (20.8)	43 (44.8)	28 (29.2)
To what extent has the consultation given you better insight into how to manage/treat nausea and vomiting in pregnancy?^a^	3 (3.8)	2 (2.6)	25 (31.7)	26 (32.9)	23 (29.1)

Participant satisfaction with the pharmacist consultation in early pregnancy, *n* = 96

^a^Answered by 79 (79/96, 82.3%) of the women who experienced nausea and vomiting in pregnancy

### Impact on quality of life

**Fig. 2:**
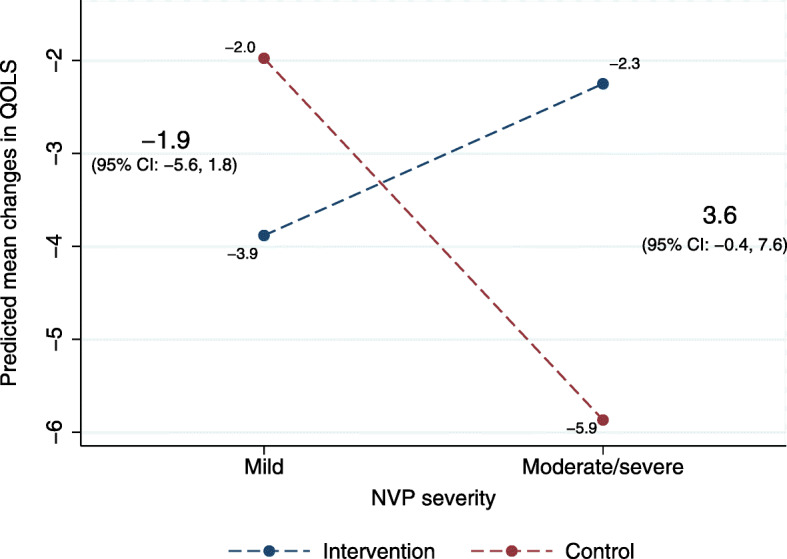
Interaction plot for the predicted mean changes in the Quality of Life Scale (QOLS) scores in the two study groups on different levels of nausea and vomiting (NVP) (*p* = 0.048). Interpretation of interaction plots: parallel lines indicate no interaction, and non-parallel lines indicate an interaction (the more non-parallel the lines are, the greater the strength of the interaction). Abbreviations: *95% CI* 95% confidence interval

The adjusted primary analysis showed that the pharmacist consultation did not have an overall impact on the women’s QOLS scores from baseline to Q2 compared to standard care (adjusted β: 0.7; 95% CI: -2.1, 3.4). The secondary stratified analyses showed that the impact of the intervention did differ according to NVP severity. While the group with no/mild NVP had scores 1.4 point lower on the QOLS after the intervention, the women with moderate/severe NVP had average scores that were 3.4 points higher compared to their respective control groups (Table 3). The interaction term between NVP severity and study group was borderline statistically significant (*p* = 0.048), indicating that the impact of the intervention on QOLS scores differed between women with no/mild NVP and women with moderate/severe NVP (Fig. 2). Results of the sensitivity analyses, according to the per-protocol principle, did not substantially differ from those of the primary and secondary analyses (Table 3 and Additional File 6).

**Table 3:** Impact of the intervention on the women's QOLS

	n	QOLS Q1	QOLS Q2	Change in QOLS Q2-Q1^a^	Change in QOLS from Q1 to Q2^a^	Change in QOLS from Q1 to Q2^a^
		Mean (range)	Mean (range)	Mean of change (range)	Crude change (β) (95% CI)	Adjusted^b^ change (β) (95% CI)
**Primary analyses**						
**Intention-to-treat**						
Intervention	119	89 (42, 108)	86 (16, 111)	-3 (-44, 40)	1.5 (-1.4, 4.5)	0.7 (-2.1, 3.4)
Control group	126	91 (62, 112)	86 (38, 111)	-4 (-58, 24)	*Reference*	*Reference*
**Secondary stratified analyses**						
**No/mild NVP**^c^						
Intervention	62	88 (43, 112)	86 (16, 111)	-3 (-44, 34)	-0.4 (-4.2, 3.3)	-1.4 (-5.1, 2.2)
Control group	69	93 (70, 111)	90 (66, 111)	-3 (-23, 22)	*Reference*	*Reference*
**Moderate/severe NVP**^c^						
Intervention	57	89 (42, 112)	87 (52, 110)	-2 (-33, 40)	3.8 (-1.0, 8.5)	3.6 (-0.6, 7.7)
Control group	57	89 (62, 112)	84 (38, 105)	-6 (-58, 24)	*Reference*	*Reference*
**Sensitivity analyses**						
**Per-protocol**						
Intervention	103	90 (43, 112)	88 (42, 111)	-3 (-38, 40)	1.6 (-1.4, 4.6)	1.2 (-1.6, 3.9)
Control group	126	91 (62, 112)	87 (38, 111)	-4 (-58, 24)	*Reference*	*Reference*

Impact of the intervention on the change in QOLS from baseline (Q1) to the second trimester (Q2)

Abbreviations: *QOLS* Quality of Life Scale; *Q1* baseline questionnaire in first trimester; *Q2* second questionnaire in second trimester; *β* beta coefficient; *95% CI* 95% confidence interval; *NVP* nausea and vomiting in pregnancy

^a^Positive score indicates improvement on the QOLS

^b^Adjusted for the QOLS score at baseline (Q1)

^c^NVP severity classified according to the Pregnancy-Unique Quantification of Emesis (PUQE) score in the baseline questionnaire (Q1): mild ≤ 6; moderate 7–12; severe ≥ 13

## Discussion

To our knowledge, this study is the first to investigate the impact of a community pharmacist consultation provided to women in early pregnancy. The results showed that the consultation did not have an overall significant impact on the women’s quality of life but highlight several important considerations regarding the pharmacists’ role in improving women’s health during gestation.

Our findings suggest that the women greatly appreciated the pharmacist consultation. The opportunity to obtain tailored information and reassurance about safe medication use in pregnancy was reported as especially useful. This finding is in line with previous studies indicating that pregnant women want to be actively involved in treatment decisions during pregnancy but that the information they receive from healthcare providers is often insufficient [8, 36]. Indeed, both professional organizations and healthcare providers have called for pharmacists to have a role in educating pregnant women and promoting safe medication use during pregnancy [37, 38]. Topics of particular importance include promoting folic acid use, adherence to needed medications, and correcting misconceptions about medicines in pregnancy [39, 40].

The results from this study further highlight NVP as a potential area where a pharmacist’s expertise may improve women’s health in antenatal care. The impact of the pharmacist consultation in this study was significantly greater among women with moderate/severe NVP when compared to those with no/mild NVP (Fig. 2), suggesting that a potential pharmacy intervention should specifically target pregnant women with more severe NVP symptoms. From a clinical point of view, it is important that these women are given information and support about NVP and NVP treatment so they can optimally manage their condition. Indeed, the importance of initiating pharmacologic antiemetic treatment when needed and taking the medications continuously is crucial for efficacy [7]. Moreover, lifestyle and dietary changes are recommended as first-line treatment for mild NVP and in addition to pharmacologic treatment for more severe symptoms [7, 41]. Community pharmacists can assess NVP severity based on the easy-to-use PUQE score and provide information based on each woman’s symptoms [32, 34]. The focus on this kind of patient-centered care has been increasing because of its benefits for quality and continuity of care [8].

All women in our study had a slight decrease in QOLS from the first to the second trimester (Table 3). The decrease in quality of life among pregnant women has also previously been reported by Chang et al. in Taiwan [42], Bai et al. in the Netherlands [43], and Morin et al. in France [44]. The observed decrease in quality of life is likely multifactorial, including both physical and emotional changes as the pregnancy proceeds. In a systematic review by Lagadec et al., it was found that women’s quality of life seems to decrease throughout pregnancy, especially quality of life related to physical functioning [1]. The authors also identified medically assisted reproduction, stress, anxiety, obstetric complications, and NVP, among other factors, to be associated with poorer quality of life during pregnancy.

Our intervention did not have an overall effect on pregnant women’s quality of life as compared to women receiving standard care. These findings should be interpreted with the nature of our study population in mind, with an overrepresentation of healthy and resourceful pregnant women with a high QOLS at baseline. Indeed, compared to the general birthing population in Norway, our study population had higher rates of women in a committed relationship and taking a folic acid supplement before and/or during pregnancy, and lower rates of smoking (Table 1). This may explain why the intervention yielded no significant overall effect on changes in QOLS scores from the first to the second trimesters. Studies have previously shown that women in a committed relationship and with higher education are more likely to seek information online compared to single women and women with a high school education or less [45]. This association may have led to that women in our study were already well informed and had the information they needed to self-manage ailments and medications during pregnancy without substantial concerns. This is also supported by the feedbacks from the participants in the intervention group, who reported that they felt reassured when healthcare personnel confirmed what they already knew about medications and treatment of ailments during pregnancy. Moreover, factors that have been associated with a better quality of life during pregnancy include primiparity, early gestational age, and having family and friends [1]. These characteristics were well represented among our study sample and further support our interpretation of the results. Because studies have repeatedly shown that patients often feel confused about their medication regimens and that the adherence for needed medications often is low [46], community pharmacists’ time and capacity would likely be best applied where the potential health benefits are the greatest: for women with more severe NVP symptoms.

In addition to the highly selected population, some other limitations should be taken into consideration when interpreting our results. First, we did not recruit our target number of participants and had a relatively high dropout rate (33.6%), as reported in Fig. 1. More than one fourth (34/124, 27.4%) of these dropouts, however, were the result of pregnancy loss, which could not be prevented. A total sample size of 245 was calculated as having 72% power to detect a 5-point difference in the QOLS scores between study groups (ad-hoc analysis). Second, we used a generic instrument, the QOLS, to assess the change in pregnant women’s quality of life from the first to the second trimester. One of the advantages of using generic instruments is that they are developed to capture the general quality of life across a heterogeneous population [30]. Because the pregnant population can consist of any women suffering from any condition, the generic approach seemed reasonable. An NVP-specific quality of life-instrument, the NVPQOL [47], and the generic Short Form Health Survey (SF-36) have, for example, been found to measure complementary aspects of health-related quality of life in a pregnant population [4]. We cannot, however, rule out that the generic instrument may not have been sensitive enough to capture certain pregnancy-specific domains concerning quality of life in this specific population [4, 48]. We recommend that future studies include an NVP-specific instrument to address this matter further. In addition, we recommend future studies to include NVP as an inclusion criterion to allow the intervention to be more targeted towards NVP.

## Conclusions

The pregnant women in this study greatly appreciated a pharmacist consultation in early pregnancy, but the intervention had no impact compared to standard care on their quality of life scores from the first to the second trimesters. Of note, a pharmacist consultation seemed to have had a greater effect for women with moderate/severe NVP compared with women with no/mild NVP. Future studies should further explore the impact of a pharmacist intervention specifically for women with NVP and on other outcomes such as the use of healthcare services and medications in pregnancy.

## Supplementary Information

**Additional file 1.** The geographical distribution of the 14 study pharmacies in Norway. Map: Google Earth.

**Additional file 2.** Norwegian treatment algorithm for nausea and vomiting in pregnancy (NVP) and hyperemesis gravidarum (HG). Adapted from the Obstetric Guideline issued by the Norwegian Society of Obstetrics and Gynecology [49].

**Additional file 3.** Flowchart of the total study sample including their nausea and vomiting in pregnancy (NVP) severity based on the Pregnancy-Unique Quantification of Emesis (PUQE) score at baseline. No/mild NVP: PUQE ≤ 6; moderate/severe NVP: PUQE = 7–15.

**Additional file 4.** Comparison of baseline characteristics of those who responded to both the baseline and the second questionnaire (complete cases) and those who dropped out of the study after the completion of the baseline questionnaire (dropouts).

**Additional file 5.** Participant satisfaction with the pharmacist consultation stratified by nausea and vomiting (NVP) severity.

**Additional file 6.** Impact of the intervention on the difference in the Quality of Life Scale (QOLS) from baseline (Q1) to the second trimester (Q2) stratified by nausea and vomiting in pregnancy (NVP) severity and according to the per-protocol principle.

## Data Availability

The dataset generated and/or analyzed during the current study are not publicly available due to the General Data Protection Regulation (GDPR) but are available from the corresponding author on reasonable request.

## Abbreviations

ADPS: Automated data preprosessing system
NVP: Nausea and vomiting in pregnancy
NVPQOL: Nausea and Vomiting in Pregnancy-specific Quality of Life-instrument
PUQE: Pregnancy-Unique Quantification of Emesis 24
QOLS: Quality of Life Scale
Q1: First online questionnaire during the first trimester
Q2: Second online questionnaire during the second trimester
SD: Standard deviation
β: Beta cofficient
95% CI: 95% cofficient
