# Supplementary figures and images for: The effect of a pharmacist consultation on pregnant women’s quality of life with a special focus on nausea and vomiting: an intervention study
Source: BMC Pregnancy Childbirth. 2020 Dec 9;20:766. doi: 10.1186/s12884-020-03472-z (PMC7727235; doi:10.1186/s12884-020-03472-z)

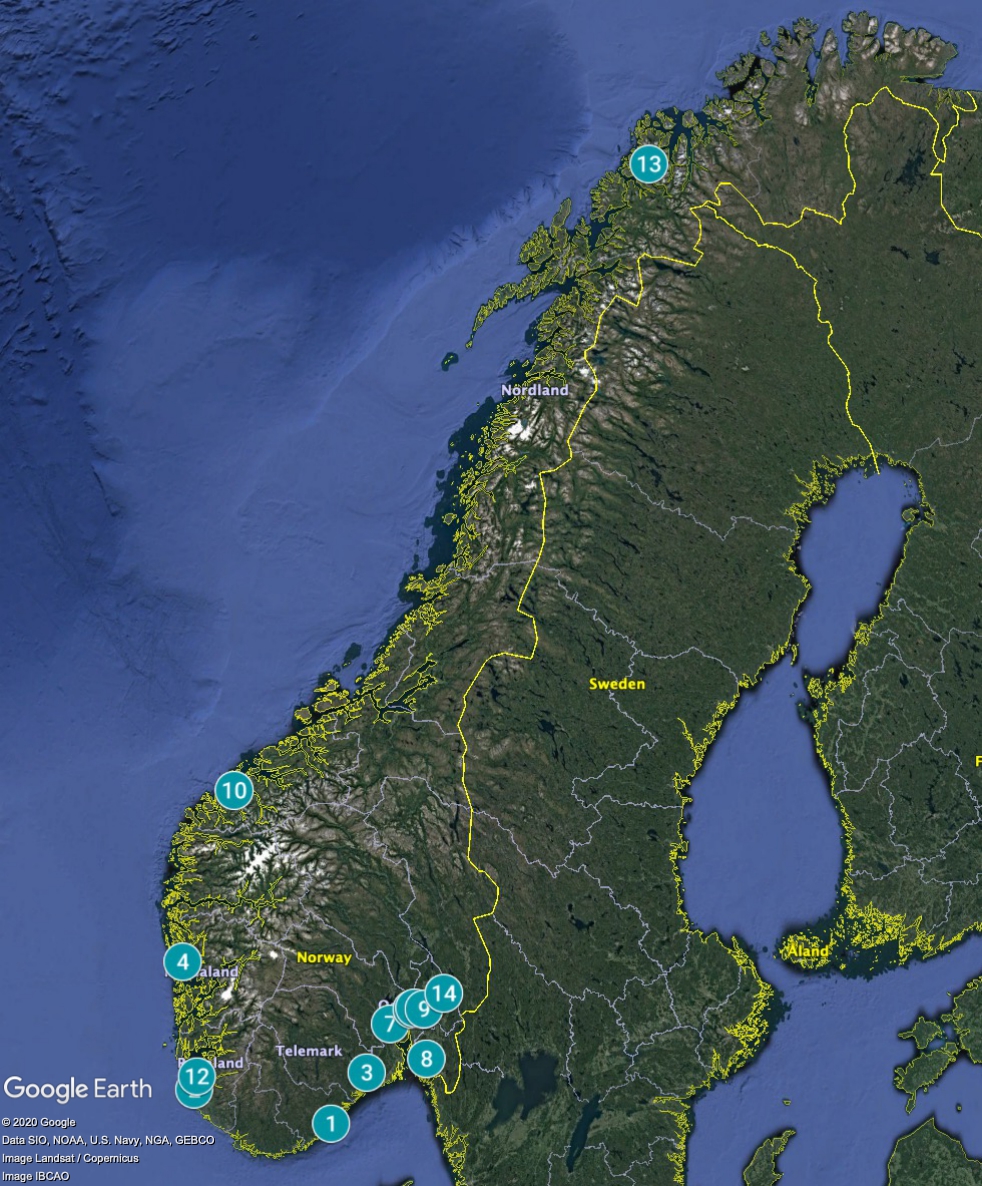

Supplement: Supplementary file 1 — Additional file 1. The geographical distribution of the 14 study pharmacies in Norway. Map: Google Earth. [file 12884_2020_3472_MOESM1_ESM.jpg]
