# Supplementary material for: The effect of a pharmacist consultation on pregnant women’s quality of life with a special focus on nausea and vomiting: an intervention study
Source: BMC Pregnancy Childbirth. 2020 Dec 9;20:766. doi: 10.1186/s12884-020-03472-z (PMC7727235; doi:10.1186/s12884-020-03472-z)
Supplement: Supplementary file 2 — Additional file 2. Norwegian treatment algorithm for nausea and vomiting in pregnancy (NVP) and hyperemesis gravidarum (HG). Adapted from the Obstetric Guideline issued by the Norwegian Society of Obstetrics and Gynecology [49]. [file 12884_2020_3472_MOESM2_ESM.pdf]

Mild NVP
PUQE score 3-12 and no complications:
- Dietary and lifestyle measures
- Antihistamines *(after initial consultation with physician)*
- Self-assess NVP symptoms 2-3 times per week
- Follow-up in community by pharmacist and/or midwife

Moderate and severe NVP
PUQE score 3-12 with complications or PUQE score ≥13:
- Dietary and lifestyle measures
- Antihistamines and/or other antiemetics
- Self-assess NVP symptoms daily
- Follow-up by physician and multidisciplinary team

HG
- In-hospital treatment
- Follow-up by specialist and multidisciplinary team

Non-pharmacological measures

Dietary recommendations
- Keep good hydration at all times: frequent fluid intake, including in between meals
- Snack before rising in the morning and get up slowly
- Small, frequent meals every 1-2 hours
- Avoid fatty/fried/acidic foods and increase proteins

Lifestyle changes
- Avoid triggers
- Get sufficient sleep and rest
- Adapt work tasks
- Reduce stress
- Get help with household chores

Complementary treatment
- **Ginger** 250 mg up to × 4
- **Acupressure/ acupuncture**

Pharmacotherapy

Vitamin B6/pyridoxine 40 mg × 2 in combination with an antihistamine
- Meclozine 25 mg × 2

or
- Doxylamine/pyridoxine 10mg/10mg delayed release combination 1+1+2 *

Insufficient effects of antihistamines on NVP symptoms:

An antiemetic from another drug class should be added.

- Promethazine 25 mg × 2-3

or
- Metoclopramide 5-10 mg × 3

or
- Prochlorperazine 5-10 mg × 2-3

- Ondansetron 4-8 mg × 3

In-hospital treatment
- IV fluids
- Any oral or IV use of medications above
- Thiamine supplementation *(required for all women admitted with prolonged vomiting)*
- Methylprednisolone 40-50 mg daily

No NVP symptoms for 1 week: discontinue treatment and assess condition

Monitor for adverse drug reactions.

Key points:
- Base treatment decisions on assessment of NVP symptoms, weight, and intake of foods and liquids.
- Assess impact of NVP symptoms on quality of life and daily functioning.
- Use PUQE scale to determine severity of NVP symptoms.
- Treat concurrent symptoms (reflux, constipation), if present.
- Multidisciplinary monitoring and follow-up.

*First-line treatment for NVP and licensed for NVP in the UK and the US.
