# Supplementary material for: The effect of a pharmacist consultation on pregnant women’s quality of life with a special focus on nausea and vomiting: an intervention study
Source: BMC Pregnancy Childbirth. 2020 Dec 9;20:766. doi: 10.1186/s12884-020-03472-z (PMC7727235; doi:10.1186/s12884-020-03472-z)
Supplement: Supplementary file 3 — Additional file 3. Flowchart of the total study sample including their nausea and vomiting in pregnancy (NVP) severity based on the Pregnancy-Unique Quantification of Emesis (PUQE) score at baseline. No/mild NVP: PUQE ≤ 6; moderate/severe NVP: PUQE = 7–15. [file 12884_2020_3472_MOESM3_ESM.pdf]

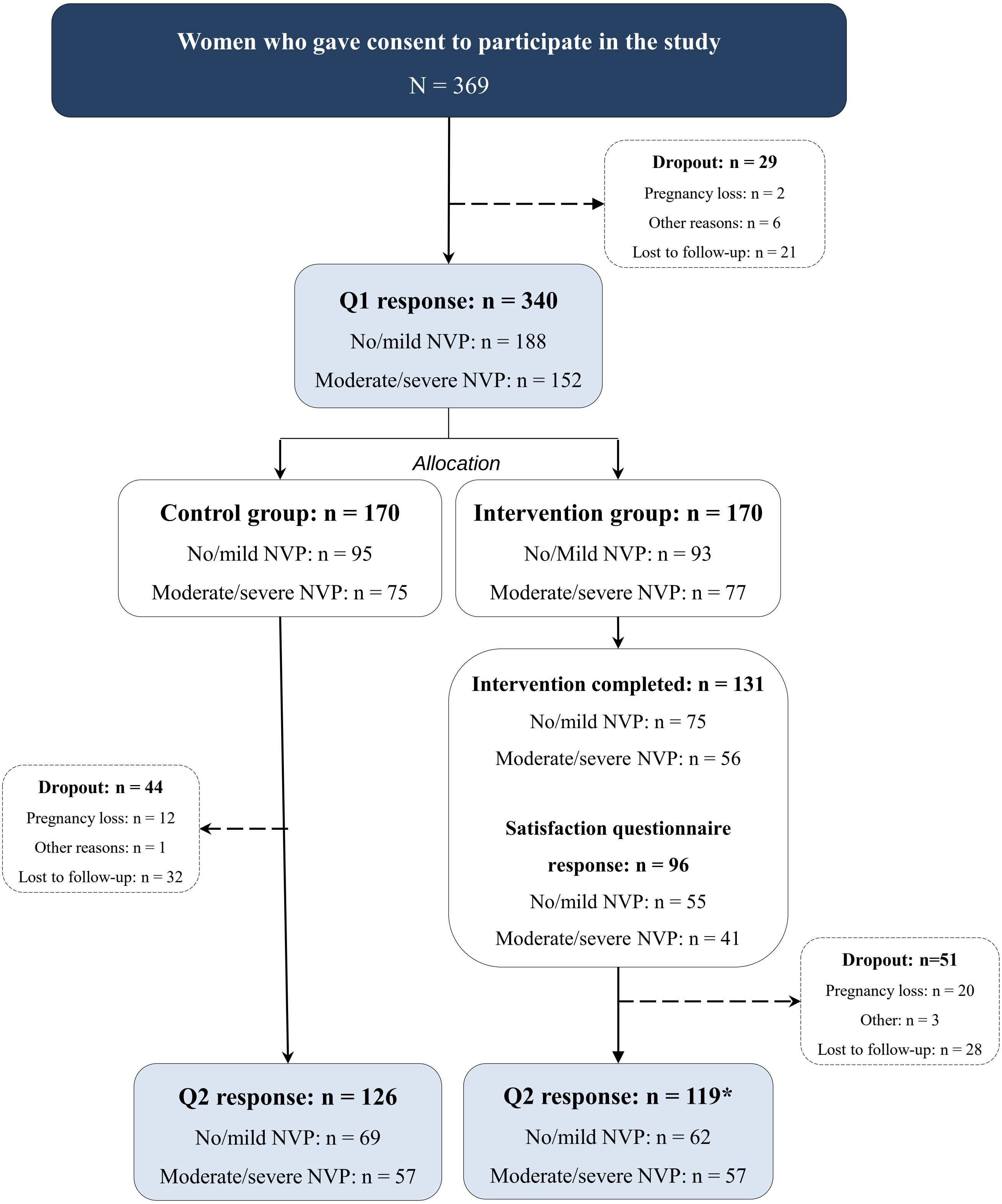
Women who gave consent to participate in the study
N = 369
Dropout: n = 29
Pregnancy loss: n = 2
Other reasons: n = 6
Lost to follow-up: n = 21
Q1 response: n = 340
No/mild NVP: n = 188
Moderate/severe NVP: n = 152
Allocation
Control group: n = 170
No/mild NVP: n = 95
Moderate/severe NVP: n = 75
Intervention group: n = 170
No/Mild NVP: n = 93
Moderate/severe NVP: n = 77
Intervention completed: n = 131
No/mild NVP: n = 75
Moderate/severe NVP: n = 56
Satisfaction questionnaire response: n = 96
No/mild NVP: n = 55
Moderate/severe NVP: n = 41
Dropout: n = 44
Pregnancy loss: n = 12
Other reasons: n = 1
Lost to follow-up: n = 32
Dropout: n=51
Pregnancy loss: n = 20
Other: n = 3
Lost to follow-up: n = 28
Q2 response: n = 126
No/mild NVP: n = 69
Moderate/severe NVP: n = 57
Q2 response: n = 119*
No/mild NVP: n = 62
Moderate/severe NVP: n = 57
