# Supplementary material for: The effect of a pharmacist consultation on pregnant women’s quality of life with a special focus on nausea and vomiting: an intervention study
Source: BMC Pregnancy Childbirth. 2020 Dec 9;20:766. doi: 10.1186/s12884-020-03472-z (PMC7727235; doi:10.1186/s12884-020-03472-z)
Supplement: Supplementary file 4 — Additional file 4. Comparison of baseline characteristics of those who responded to both the baseline and the second questionnaire (complete cases) and those who dropped out of the study after the completion of the baseline questionnaire (dropouts). [file 12884_2020_3472_MOESM4_ESM.docx]

Additional File 4 Comparison of baseline characteristics between the analyzed sample (complete cases) and those who dropped out.

| **Baseline characteristics** | **Complete cases**^*^**,**  n = 245 | **Dropouts**^*^**,**  n = 95 |
| --- | --- | --- |
| Gestational week, mean (SD, range) | 7.6 (2.3, 3-12) | 7.1 (2.4, 3-12) |
| Maternal age, mean (SD, range) | 31.2 (4.3, 18-44) | 31.0 (4.0, 22-41) |
| Primiparous, % | 53.5 | 51.6 |
| University/college degree, % | 82.9 | 76.8 |
| Employed, % | 85.3 | 85.3 |
| Married/cohabitant, % | 96.7 | 97.9 |
| ≥1 chronic condition, % | 44.5 | 49.5 |
| PUQE score^a^, mean (SD, range) | 6.4 (0.2, 3-15) | 6.0 (0.2, 3-12) |
| Moderate or severe NVP^a^, % | 46.5 | 40.0 |
| Infertility treatment in current pregnancy, % | 13.9 | 11.6 |
| Folic acid supplement before and during pregnancy, % | 98.0 | 97.9 |
| Smoking in pregnancy, % | 1.2 | 1.1 |
| Alcohol in pregnancy, % | 4.1 | 2.1 |
| QOLS, mean (SD, range) | 90.3 (11.4, 42-112) | 89.3 (11.4, 48-112) |
| Intervention group, % | 48.6 | 53.7 |

**Comparison of baseline characteristics of those who responded to both the baseline and the second questionnaire (complete cases) and those who dropped out of the study after the completion of the baseline questionnaire (dropouts).**

Abbreviations: **SD:** standard deviation; **NVP**: nausea and vomiting in pregnancy; **QOLS**: Quality of Life Scale.

^*^The Chi-Squared test was used to compare categorical variables, and the two-sided Students t-test were used for continuous variables (gestation week, maternal age, PUQE, and QOLS). No results were statistically significant, i.e., all p ≥ 0.05.

^a^NVP severity based on the Pregnancy-Unique Quantification of Emesis (PUQE) score: mild ≤ 6; moderate 7-12; severe ≥ 13.
