# Supplementary material for: The effect of a pharmacist consultation on pregnant women’s quality of life with a special focus on nausea and vomiting: an intervention study
Source: BMC Pregnancy Childbirth. 2020 Dec 9;20:766. doi: 10.1186/s12884-020-03472-z (PMC7727235; doi:10.1186/s12884-020-03472-z)
Supplement: Supplementary file 5 — Additional file 5. Participant satisfaction with the pharmacist consultation stratified by nausea and vomiting (NVP) severity. [file 12884_2020_3472_MOESM5_ESM.docx]

**Additional File 5** Participant satisfaction with the pharmacist consultation stratified by nausea and vomiting (NVP) severity^a^.

|  | **Total**  **n = 96** | **Very little** | | **Little** | | **Neutral** | | **Large** | | **Very large** |  |
| --- | --- | --- | --- | --- | --- | --- | --- | --- | --- | --- | --- |
|  | n | n (%) | | n (%) | | n (%) | | n (%) | | n (%) | p |
| In total, to what extent are you satisfied with the consultation you were provided?  No/mild NVP  Moderate/severe NVP | 55  41 | 0 (0.0)  0 (0.0) | | 0 (0.0)  1 (2.4) | | 4 (7.3)  2 (4.9) | | 22 (40.0)  20 (48.8) | | 29 (52.7)  18 (43.9) | 0.50 |
| To what extent did you find the consultation useful?  No/mild NVP  Moderate/severe NVP | 55  41 | 1 (1.8)  0 (0.0) | | 1 (1.8)  2 (4.9) | | 9 (16.4)  5 (12.2) | | 28 (50.9)  22 (53.7) | | 16 (29.1)  12 (29.3) | 0.78 |
| To what extent was the consultation worth the time spent?  No/mild NVP  Moderate/severe NVP | 55  41 | 1 (1.8)  0 (0.0) | | 2 (3.6)  1 (2.4) | | 7 (12.7)  5 (12.2) | | 26 (47.3)  21 (51.2) | | 19 (34.6)  14 (34.2) | 0.92 |
| To what extent was the pharmacist concerned about you and your pregnancy?  No/mild NVP  Moderate/severe NVP | 55  41 | 12 (21.8)  2 (4.9) | | 0 (0.0)  2 (4.9) | | 6 (10.9)  9 (22.0) | | 24 (43.6)  19 (46.3) | | 13 (23.6)  9 (22.0) | 0.06 |
| To what extent has the consultation given you better insight into how to use medications during pregnancy?  No/mild NVP  Moderate/severe NVP | 55  41 | 0 (0.0)  0 (0.0) | | 0 (0.0)  1 (2.4) | | 8 (14.6)  4 (9.8) | | 15 (27.3)  16 (39.0) | | 32 (58.2)  20 (48.8) | 0.37 |
| To what extent did you find a solution to your problems/concerns?  No/mild NVP  Moderate/severe NVP | 55  41 | 2 (3.6)  0 (0.0) | | 0 (0.0)  3 (7.3) | | 13 (23.6)  7 (17.1) | | 24 (43.6)  19 (46.3) | | 16 (29.1)  12 (29.3) | 0.20 |
| To what extent has the consultation given you better insight into how to manage/treat nausea and vomiting in pregnancy?^b^  Mild NVP  Moderate/severe NVP | 38  41 | 2 (5.6)  1 (2.4) | 1 (2.6)  1 (2.4) | | 11 (29.0)  14 (34.2) | | 13 (24.6)  13 (31.7) | | 11 (29.0)  12 (29.3) | | 0.96 |

Participant satisfaction with the pharmacist consultation in early pregnancy stratified by nausea and vomiting (NVP) severity.

^a^NVP severity classified according to the Pregnancy-Unique Quantification of Emesis (PUQE) score in the baseline questionnaire (Q1): mild ≤ 6; moderate 7-12; severe ≥ 13.

^b^Answered by 79 (79/96, 82.3%) of the women who experienced NVP.
