# Supplementary material for: The effect of a pharmacist consultation on pregnant women’s quality of life with a special focus on nausea and vomiting: an intervention study
Source: BMC Pregnancy Childbirth. 2020 Dec 9;20:766. doi: 10.1186/s12884-020-03472-z (PMC7727235; doi:10.1186/s12884-020-03472-z)
Supplement: Supplementary file 6 — Additional file 6. Impact of the intervention on the difference in the Quality of Life Scale (QOLS) from baseline (Q1) to the second trimester (Q2) stratified by nausea and vomiting in pregnancy (NVP) severity and according to the per-protocol principle. [file 12884_2020_3472_MOESM6_ESM.docx]

Additional File 6 Impact of the intervention on QOLS scores according to the per-protocol-principle.

| **NVP severity**^1^ | n | **QOLS**  **Q1** | **QOLS**  **Q2** | **Change in QOLS**  **Q2-Q1**^2^ | **Change in QOLS from Q1 to Q2**^2^ | **Change in QOLS from Q1 to Q2**^2^ |
| --- | --- | --- | --- | --- | --- | --- |
|  |  | Mean (range) | Mean (range) | Mean of diff. (range) | Crude change (β) (95% CI) | Adjusted^3^ change (β) (95% CI) |
| **Mild**  Intervention  Control | 54  69 | 91 (43, 112)  93 (70, 111) | 87 (42, 111)  90 (66, 111) | -3 (-38, 34)  -3 (-23, 22) | -0.3 (-3.8, 3.3)  *Reference* | -0.9 (-4.2, 2.4)  *Reference* |
| **Moderate/severe**  Intervention  Control | 49  57 | 90 (59, 112)  89 (62, 112) | 88 (62, 110)  84 (38, 105) | -2 (-33, 40)  -6 (-58, 24) | 3.7 (-1.3, 8.7)  *Reference* | 3.9 (-0.4, 8.2)  *Reference* |

**Impact of the intervention on the difference in QOLS from baseline (Q1) to the second trimester (Q2) stratified on NVP severity according to the per-protocol-principle.**

Abbreviations: **QOLS:** Quality of Life Scale; **Q1:** baseline questionnaire in first trimester; **Q2:** second questionnaire in second trimester; **β:** beta coefficient; **95% CI:** 95% confidence interval; **NVP:** nausea and vomiting in pregnancy.

^1^NVP severity classified according to the Pregnancy-Unique Quantification of Emesis (PUQE) score in the baseline questionnaire (Q1): mild ≤ 6; moderate 7-12; severe ≥ 13.

^2^Positive score indicates improvement in the Quality of Life Scale.

^3^Adjusted for the QOLS score at baseline (Q1).
